# G-Quadruplexes in Human Telomere: Structures, Properties, and Applications

**DOI:** 10.3390/molecules29010174

**Published:** 2023-12-27

**Authors:** Yan Xu, Makoto Komiyama

**Affiliations:** 1 Division of Chemistry, Department of Medical Sciences, Faculty of Medicine, University of Miyazaki, 5200 Kihara, Kiyotake, Miyazaki 889-1692, Japan; 2 Research Center for Advanced Science and Technology (RCAST), The University of Tokyo, 4-6-1 Komaba, Meguro, Tokyo 153-8904, Japan

**Keywords:** DNA G-quadruplex, RNA G-quadruplex, DNA:RNA hybrid G-quadruplex

## Abstract

G-quadruplexes, intricate four-stranded structures composed of G-tetrads formed by four guanine bases, are prevalent in both DNA and RNA. Notably, these structures play pivotal roles in human telomeres, contributing to essential cellular functions. Additionally, the existence of DNA:RNA hybrid G-quadruplexes adds a layer of complexity to their structural diversity. This review provides a comprehensive overview of recent advancements in unraveling the intricacies of DNA and RNA G-quadruplexes within human telomeres. Detailed insights into their structural features are presented, encompassing the latest developments in chemical approaches designed to probe these G-quadruplex structures. Furthermore, this review explores the applications of G-quadruplex structures in targeting human telomeres. Finally, the manuscript outlines the imminent challenges in this evolving field, setting the stage for future investigations.

## 1. Introduction

**Figure 1:**
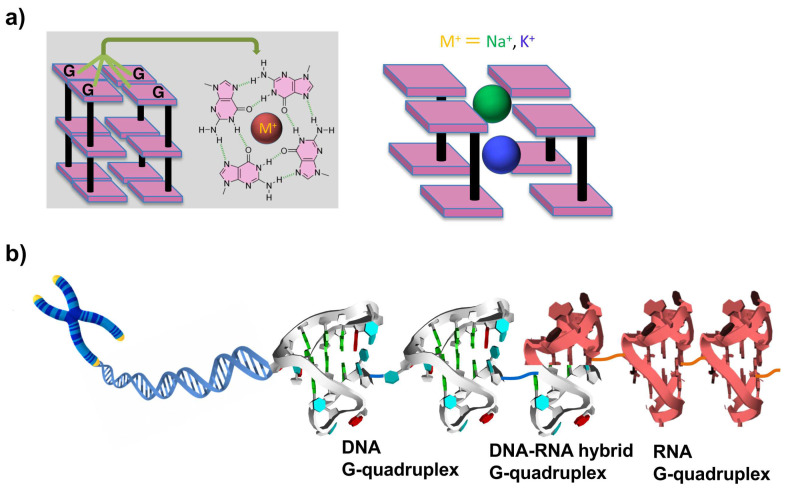
(**a**) Structures of G tetrads and G-quadruplexes. Cations with larger ionic radii, such as K^+^ (depicted in blue), are positioned between the tetrads. In contrast, smaller cations, such as Na^+^ (illustrated in green), can coordinate within the plane of the tetrads or assume an intermediate position. (**b**) Superhelix structure formed at the ends of chromosomes from telomeric DNA G-quadruplexes, DNA:RNA G-quadruplexes, and RNA G-quadruplexes. As the result, this essential portion is sufficiently protected and further functionalized.

The term telomere is derived from two Greek words: telos (meaning ‘‘end’’) and meros (meaning ‘‘part’’). Human telomeric DNA consists of a duplex region composed of TTAGGG repeats, which terminates with a G-rich single-stranded overhang of 100–200 nucleotide (nt) length. Though telomeres are crucial for many cellular functions, they are subject to progressive decreases in length at each round of DNA replication. It has been demonstrated by many studies that human telomere DNA plays important roles in cancer and aging [1,2]. Telomeres provide the machinery to protect chromosomes from such detriments. The protective function of telomeres might depend on their state, e.g., whether they have ‘‘capping’’ or ‘‘uncapping’’ structures. Telomere DNA sequences can form G-quadruplexes with various folding topologies, as demonstrated by many structural studies [1]. As shown in Figure 1a, four guanine bases are bound to a tetrad through Hoogsteen hydrogen bondings, and these guanine tetrads stack each other into G-quadruplexes. Metal cations (e.g., Na^+^ and K^+^) are usually bound to the center of guanine tetrad to stabilize the whole structures. G-quadruplexes are promising for numerous approaches that were developed to target telomeres [3,4,5]. Several ligands for G-quadruplexes were developed, and their interactions were investigated in detail. For a long time, telomere DNA was believed not to be transcribed into RNA. However, more recent findings showed that telomere DNA is in fact transcribed into RNA (referred to as telomere RNA) in cells [6,7]. These telomere RNAs exist in human cells with heterogeneous length. It was proposed that telomere RNA is associated with the end chromosome, and reveals a new level of protection of chromosomal ends [8,9,10]. Valuable insight into biological processes such as cancer and aging has been provided.

Recently, telomere RNA was suggested to form G-quadruplexes which perform complicated functions [11,12,13,14]. With the use of probes provided by chemical approaches, telomere DNA and RNA G-quadruplex structures were detected in vitro and also in human cells [15,16,17]. Moreover, an DNA:RNA hybrid G-quadruplex is formed from telomeric DNA and telomeric RNA [18,19,20,21,22]. These results indicate that DNA G-quadruplexes, DNA:RNA hybrid G-quadruplexes, and RNA G-quadruplexes can be key players to protect the terminus of telomere (Figure 1b).

In order to deeply understand the biology of telomere, precise information on the structures of G-quadruplexes and their physicochemical properties is essential. Accordingly, the present review focuses on the structural information of DNA G-quadruplexes, RNA G-quadruplexes, and DNA:RNA hybrid G-quadruplexes in human telomere. Various chemical approaches to probe human telomere G-quadruplex structures, as well as applications of G-quadruplex structures to target human telomere, are also described. Lastly, the future aspects of these unique noncanonical structures are discussed.

## 2. DNA G-Quadruplexes in Human Telomere

**Figure 2:**
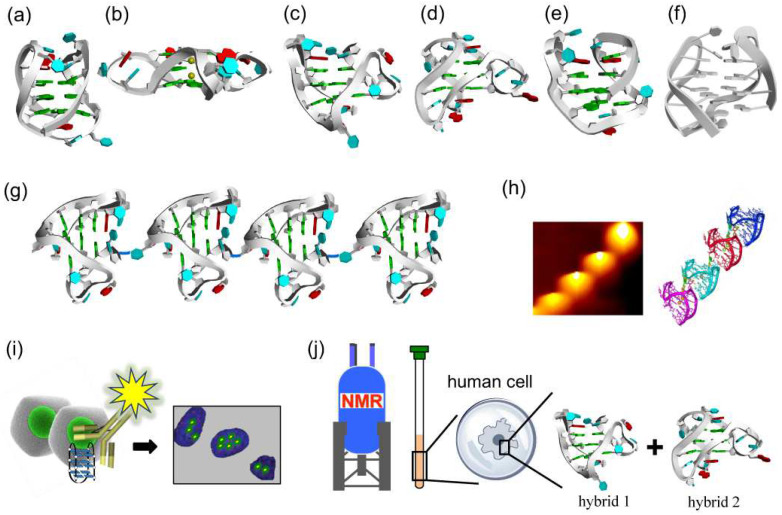
Variety of conformations of DNA G-quadruplexes observed under different conditions. (**a**) Antiparallel G-quadruplex from d[AGGG(TTAGGG)_3_] formed in Na^+^ solution. (**b**) A crystal structure from d[AGGG(TTAGGG)_3_] in the presence of K^+^ ions. (**c**,**d**) Hybrid-1 type and hybrid-2 type G-quadruplexes from d[AGGG(TTAGGG)_3_] prepared in K^+^ solution. (**e**) Antiparallel G-quadruplex of d[GGG(TTAGGG)_3_T] with only two G-tetrad layers in K^+^ solution. (**f**) Chair antiparallel conformation from d[(GGGTTA)_2_GGGTTTGGG] in K^+^ solution. (**g**,**h**) Higher order G-quadruplex observed by AFM. (**i**) Visualization of DNA G-quadruplex structures in cells by immunostaining with the use of structure-specific antibody. (**j**) In-cell ^19^F NMR of d[AGGG(TTAGGG)_3_] in Hela cells.

The telomeres of human chromosome ends are composed of telomeric repeats of 5′-TTAGGG-3′ sequence. Through many structural studies on the human telomere, a large number of G-quadruplexes of different structures have been already identified, as depicted in Figure 2 [23,24,25,26,27,28,29,30,31,32]. These structural differences are primarily based on the syn/anti conformations of guanine residues and the relative orientation of the G-quartet core [33]. Thus, the topologies of DNA G-quadruplex are drastically dependent on the sequences and lengths of telomere DNA, the types of linking loops, and the nature of the associated metal cations.

For example, in NaCl solution, a 22-mer telomeric DNA d[AGGG(TTAGGG)_3_] forms an antiparallel G-quadruplex with anti/syn guanines (Figure 2a) [34]. One loop is diagonal, and two other loops are lateral. In KCl solution, however, this sequence forms the mixtures of hybrid-1 and hybrid-2 G-quadruplexes, in which the loop types are external, lateral, and lateral (Figure 2c,d) [35,36,37,38]. In the crystal grown in the presence of K^+^ ion, the same sequence adopts a completely different parallel G-quadruplex conformation having three external linking loops (Figure 2b) [39]. On the other hand, another telomeric sequence d[GGG(TTAGGG)_3_T] forms in KCl solution an antiparallel G-quadruplex with only two G-tetrad layers (Figure 2e) [40,41,42]. More recently, d[(GGGTTA)_2_GGGTTTGGG] sequence was reported to take another antiparallel structure, so-called ‘‘chair’’ with lateral loops (Figure 2f) [43]. Circular dichroism (CD) spectroscopy is very useful to distinguish G-quadruplex structures in terms of fundamental characteristic bands [44]. For example, the CD spectrum of human telomeric sequence d[AGGG(TTAGGG)_3_] in 100 mM Na^+^ solution exhibits a 295 nm positive band and a 265 nm negative band, which is characteristic of an antiparallel G-quartet structure [35]. In a 100 mM K^+^ ion solution, however, this sequence shows a stronger positive CD band at 290 nm, with weak negative peaks near 255 and 235 nm, indicating that a mixture of hybrid G-quadruplexes is formed under these conditions. 

For the analysis of the structures of G-quadruplexes from still longer telomeric sequence, atomic force microscope (AFM) was employed (Figure 2g,h) [45]. In the AFM image, a telomere DNA (around 100-nucleotide length) forms a higher-order assembly of G-quadruplex, in which adjacent G-quadruplex conformation units are arranged in end-to-end structure. These G-quadruplex units stack each other into higher-order packing structures. Some small molecules and antibodies were also used to investigate the DNA G-quadruplexes in living cells (Figure 2i) [46,47]. 

The topology of G-quadruplex of telomeric DNA in living human cells has not yet been sufficiently clarified. To shed light on this problem, in-cell ^19^F NMR experiments were performed in the environment close to the inside of human cells. ^19^F NMR spectroscopy is suitable for this purpose, since it is characterized by (i) simplicity of spectra, (ii) high sensitivity of chemical shift to the environment, and (iii) absence of natural background signals in cells. For example, 3,5-bis(trifluoromethyl)phenyl moiety was connected to the 5′-end of telomeric DNA sequence (d[AGGG(TTAGGG)_3_]), and this ^19^F-labelled DNA was injected into human cells. According to in-cell ^19^F NMR, this telomeric DNA forms the mixture of hybrid-1 and hybrid-2 G-quadruplexes in the human cells (Figure 2j) [48,49]. In *Xenopus laevis* oocytes, a hybrid-2 type G-quadruplex is formed [50]. The two-tetrad G-quadruplex in Figure 2e was further examined by ^19^F NMR spectroscopy. Only one ^19^F NMR signal was observed both in K^+^ solution and in HeLa cells [48]. These results provide valuable information on the understanding of the structures of human telomeric DNA in living human cells and also on the design new drugs that target telomeric DNA.

Many fluorescent probes to detect DNA G-quadruplexes have been reported, and employed to image living cells [51,52,53,54,55,56,57]. In 2015, naturally occurring alkaloid epiberberine was found to emit fluorescence upon the binding with DNA G-quadruplex [58]. Later, various fluorescent probes were developed [59,60,61,62], and used to construct quadruplex-based analytical sensing platforms. Among these probes, a tripodal cationic fluorescent probe was featured by notable differences in fluorescence lifetime between G-quadruplexes and other DNA topologies [63]. Furthermore, its fluorescence quantum yield was greatly increased upon G-quadruplex binding. These two features allowed a clearcut differentiation of DNA G-quadruplexes from background signals in cell imaging, and a quantitative analysis was accomplished in terms of photon counts. According to NMR spectroscopy, this probe interacts with G-quadruplexes using three arms through π–π stacking. An artificial protein of 6.7 kD is useful to detect G-quadruplexes in living cells with the ChIP-Seq technique [64]. In this protein probe, two identical G4-binding domains (23-amino acid residues each) are connected with linker peptide to bind G-quadruplexes with sufficient strength. Small molecular ligands, which distinguish different conformations of G-quadruplexes each other, were also reported [65,66,67]. 

It is noteworthy that small molecules targeting G-quadruplexes are also important as potent drugs for therapy of cancer and other diseases [68,69,70,71,72,73,74,75,76,77,78,79]. For example, a small molecule, phenyl-ethenyl-quinoline, specifically binds G-quadruplex in oncogene MYC promoter, and is promising as a lead compound of anticancer drug [80]. This compound consists of phenyl and quinoline moieties linked via rather flexible ethenyl bridge, and has +1 charge on the methylated N1 atom. In addition to its direct interaction with the G-quadruplex core, this molecule additionally interacts with the 3′- and the 5′-flanking residues of target G-quadruplex that are immediately adjacent to the G-core. These interactions help this binder to distinguish the MYC promoter G-quadruplex from other G4 structures, as proposed previously [81]. Important roles of structured water in these G-quadruplex-small molecule interactions have been also indicated [82].

## 3. RNA G-Quadruplexes in Human Telomere

**Figure 3:**
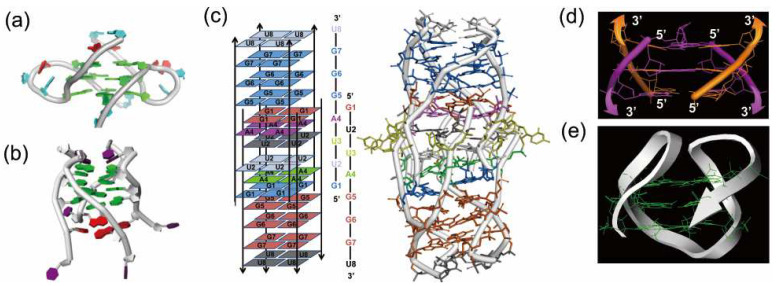
(**a**) Dimeric parallel telomeric RNA G-quadruplex from r(UAGGGUUAGGGU). (**b**) Tetramolecular RNA G-quadruplex from r(UAGGGU) possessing a novel U-tetrad on the bottom (in red). (**c**) RNA G-quadruplex having 14 tetrads, in which an eight-stranded helix part intercalates at the center. (**d**) Antiparallel RNA G-quadruplex from the fragments having 8-bromoguanosine. (**e**) Antiparallel RNA G-quadruplex formed by a 22-mer telomeric RNA.

Telomere DNA is transcribed into telomeric RNA (UUAGGG repeat) which shows various biological functions [6,7]. Importantly, RNA G-quadruplexes in most cases take parallel conformation. For example, a 12-mer telomeric RNA r(UAGGGUUAGGGU) forms a parallel dimer G-quadruplex in Na^+^ solution, as determined by ^1^H-NMR (Figure 3a) [83]. Both in K^+^ solution and in K^+^-stabilized crystal, this 12-mer RNA also forms a G-quadruplex of the same topology [12,14]. This feature is highly in contrast with the results for DNA G-quadruplexes, in which the structures enormously depend on the conditions (Figure 2). Stereochemical constraints inherent to RNA should be one of the governing factors for this difference. Very interestingly, the formation of parallel G-quadruplex from four strands of r(UAGGGU) is accompanied by simultaneous formation of a U-tetrad [13]. As shown in Figure 3b, this U-tetrad (in red), formed through Hoogsteen bindings of four uridines at the 3′-end of each RNA strand, sits on the parallel G-quadruplex structure (in green). It is noteworthy that this U-tetrad dramatically stabilizes the whole structure of RNA assembly. In cells, this kind of U-tetrad could be also formed, and promote the functions of human telomere. In many biological regulations by RNA G-quadruplex, specific proteins bind to this noncanonical structure. For example, heterogeneous nuclear ribonucleoprotein A1 (hnRNPA1) cooperates with RNA G-quadruplex to facilitate telomere capping [84,85,86]. The loops of G-quadruplex are primarily responsible for the interactions with hnRNPA1. 

Until very recently, it was believed that almost all natural RNAs form only parallel G-quadruplexes [87,88]. However, it was evidenced that an antiparallel G-quadruplex is formed in Na^+^ solution from a 22-mer RNA of telomere sequence (Figure 3e) [89]. An antiparallel G-quadruplex was also obtained in K^+^ solution, when 6-mer RNA (r(UAGGGU)) was modified with 8^Br^rG (Figure 3d) [90]. On the other hand, 8-mer telomere RNA GUUAGGGU forms an unusual RNA architecture which involves 14 tetrads such as A-, U- and G-tetrad (Figure 3c) [91]. A central scaffold with eight stranded helices intercalates two symmetry subunit G-quadruplexes. This conformation is stable and is retained even under denaturing conditions. Apparently, the structures of RNA G-quadruplexes are more complicated than expected before [92]. 

Formation of RNA G-quadruplexes in vivo was first proposed in terms of the dimerization of two genomic RNA copies of HIV through RNA-RNA interactions [93]. This dimerization was dramatically stabilized by K^+^ cation. This result, as well as other evidence, indicated the formation of an intermolecular RNA G-quadruplex, in which four guanine bases are assembled to tetrad as already known in DNA G-quadruplexes [87,94,95,96]. Later, RNA G-quadruplexes were found in many biological systems, and their essential roles were evidenced in many bioprocesses: regulation of mRNA translation [97,98,99,100,101,102,103], telomere homeostasis [104], mRNA splicing [105,106,107], and others [106,108]. Of course, their relevance to diseases is apparent [109,110]. In RNA viruses, G-quadruplexes in their genomes control viral replication, transcription, and other processes. For example, there exist many G-quadruplex folding sequences in the open reading frame regions of SARS-CoV-2 (the virus of COVID-19) [111], which caused a terrible pandemic from 2019 to 2023. Direct interaction of one of these G-quadruplexes with viral helicase, which is inevitable enzyme for the viral functions, was proposed.

**Figure 4:**
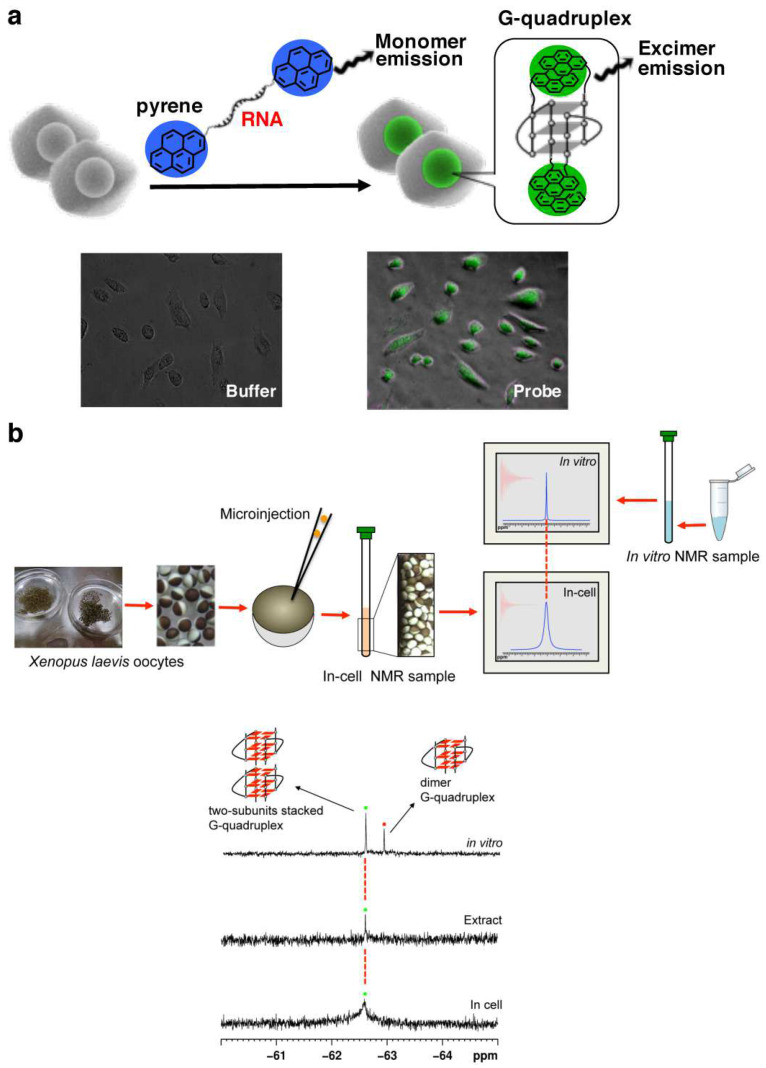
(**a**) A pyrene probe used to detect in-cell formation of G-quadruplex from telomeric RNA (r(GGGUUAGGG)). Fluorescence microscopy images of living cells with or without the probe are shown below. (**b**) In-cell ^19^F NMR experiment in *Xenopus* oocytes. The spectra of (**top**) RNA telomeric G-quadruplex in vitro and (**bottom**) in *Xenopus* oocytes.

In order to detect the formation of RNA G-quadruplex in cells, a light-switching probe was fabricated by incorporating pyrenes into both the 5′- and the 3′-ends of r(GGGUUAGGG) (Figure 4a) [17]. When G-quadruplex is formed from this RNA, the two pyrene chromophores are placed in close proximity to form their exciplexes. As the results, the maximum of fluorescence emission shifts from 400 nm (blue emission) to 480 nm (green emission). The system is employable for in-cell detection, as depicted in fluorescence microscopy images in the bottom. Furthermore, ^19^F NMR was successfully used to investigate the structure of telomeric RNA in cells (Figure 4b) [112,113,114,115,116,117]. Telomeric RNA r(UAGGGUUAGGU) was labeled with 3,5-bis(trifluoromethyl)phenyl moiety, and injected into *Xenopus laevis* oocytes [112]. A new ^19^F-NMR signal was observed (the bottom spectrum), and assigned to stacking assembly of two G-quadruplex units with the use of the reference spectrum obtained in vitro (the top).

Alternatively, RNA G-quadruplexes were detected in cells by using a coumarin-hemicyanine fluorophore (QUMA-1), which was prepared through the condensation of a coumarin aldehyde and an N-methylated quinoline derivative [118]. Its fluorescence emission at 660 nm is only weak in buffer, but is significantly enhanced by RNA G-quadruplexes. This enhancement is specific to G-quadruplexes, and either single-stranded RNA or double-stranded RNA hardly affects the emission intensity. This red-emitting fluorescent probe was successfully employed for selective, continuous, and real-time visualization of RNA G-quadruplexes in living cells. It was proposed that the tight binding of the probe to RNA G-quadruplexes restricts the rotation around the methine bridge between the coumarin and the N-methylated quinoline moiety, and locks the probe in its fluorescently active conformation. In another probe PyroTASQ, four guanines are attached to a pyrene as fluorescent chromophore [119]. The intrinsic fluorescence from this probe is minimal, since the emission from the pyrene chromophore is quenched by their own guanines throu©h intramolecular photo-induced electron transfer. Upon binding with G-quadruplex, however, this probe achieves a conformational switch to assemble its four guanines into an intramolecular G-quartet, which stacks on the analyte G-quadruplex. As a result, the electrons in the guanines are redistributed to suppress the fluorescence quenching, and the emission from the pyrene is restored. These two fluorescent probes are highly promising to sensitively detect G-quadruplexes in vivo and in vitro, since they light up only with the coexistence of G-quadruplexes and are otherwise virtually silent.

Diastereomerically pure Fe(II) metametallohelices strongly bind the G-quadruplex of human telomeric repeat-containing RNA (TERRA rG4), and stabilize this non-canonical structure [120]. Accordingly, DNA synthesis on RNA template containing four repeats of human telomeric sequence is inhibited by them. As tailor-made binders, unique aptamers towards TERRA rG4 were prepared using unnatural L-RNA units (mirror image of natural D-RNA nucleotides) [121]. Strong and specific binding of TERRA rG4 by the aptamer was evidenced. Notably, they recognized the target TERRA rG4 very strictly, and never bound other forms of constructs. Neither simple RNA hairpin nor telomeric DNA G-quadruplex was bound, and still more importantly, even other rG4s (hTERC, NRAS, miRNA149, and others) were not bound either. Cationic porphyrins (CPs) bearing an alkyl chain was used for photodynamic therapy of cancer [122]. The porphyrin derivatives preferentially bind to the RNA G-quadruplex formed in mRNA, and destroys this mRNA upon photoirradiation. 

## 4. DNA:RNA Hybrid G-Quadruplexes in Human Telomere

**Figure 5:**
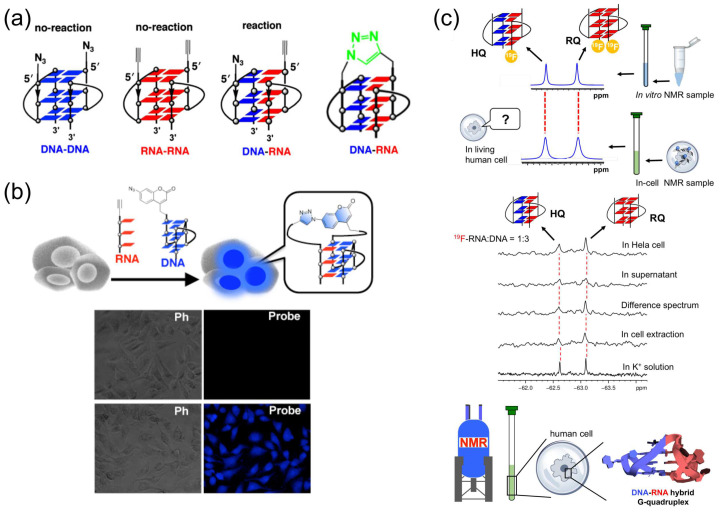
(**a**) Formation of DNA:RNA hybrid G-quadruplex promotes a click reaction to differentiate this hybrid G-quadruplex from DNA G-quadruplex and RNA G-quadruplex. (**b**) Fluorescence images with and without the probe in cells. Formation of DNA:RNA hybrid G-quadruplex in cells was confirmed. Upper images; without Cu catalyst for the click reaction. Bottom images; with Cu catalyst. Ph; phase-contrast imaging. (**c**) ^19^F NMR showing the formation of DNA:RNA hybrid G-quadruplex in human cells.

The discovery of telomere RNA soon raised a crucial question of how telomeric RNA is specifically associated with telomeric DNA to accomplish various functions (chromosome-end regulation, protection, and others). An association between telomere RNA and telomere DNA through the formation of an intermolecular DNA:RNA G-quadruplex may be an answer to this problem. However, it is technically difficult to study the DNA:RNA hybrid G-quadruplex structure by traditional methods, such as NMR spectroscopy and X-ray crystallography, since the DNA G-quadruplex, RNA G-quadruplex, and DNA:RNA hybrid G-quadruplex may coexist as a mixture in solution. Thus, concrete evidence for the formation of DNA:RNA G-quadruplex is hard to obtain. Accordingly, the experiments in Figure 5a,b were designed to trap DNA:RNA hybrid G-quadruplex by click reaction and produce a snapshot of the inter-converting structures that are present in complicated solutions. Human telomeric DNA and RNA are modified with either an alkyne or an azide, and mixed each other. The detection of ‘‘azide-alkyne cycloaddition’’ product between the DNA and the RNA is used as the evidence for the formation of DNA:RNA hybrid G-quadruplex. In Figure 5a, an alkyne is bound to the 5′-end of RNA, and an azide is bound to the 5′-end of DNA [19]. Thus, only when the DNA:RNA hybrid G-quadruplex is formed in the reaction mixture, the alkyne and the azide are placed nearby and the click reaction between them should efficiently proceed [123]. In fact, it was clearly shown by gel-electrophoresis that DNA:RNA hybrid G-quadruplex is formed from telomere DNA and telomeric RNA in aqueous K^+^ solutions [19,20,45,123]. Furthermore, this method was applied to in-cell reactions, where the DNA was labelled with an azide-bearing profluorophore (7-azidocoumarin), and incorporated into human cells together with the alkyne-bearing RNA. Direct evidence for the formation of DNA:RNA hybrid G-quadruplexes in cells was provided as fluorescent signals in the bottom images in Figure 5b [20,21,123]. Consistently, no fluorescent signals were detected in the absence of Cu catalysts for the click reactions (upper images).

With the use of ^19^F NMR spectrum, the formation of DNA:RNA hybrid G-quadruplex in living human cells was also assessed and strongly confirmed [22]. In Figure 5c, a ^19^F-labelled 12-mer G-rich RNA was mixed with 12-mer G-rich DNA in 1:3 ratio. Among two major peaks, the signal at −62.61 ppm was assigned to the DNA:RNA hybrid G-quadruplex (HQ). As shown in the top spectrum, this signal was clearly observed in living human cells, confirming the formation of this DNA:RNA hybrid G-quadruplex. Concurrently, the RNA G-quadruplex (RQ) was also formed.

Telomere RNA plays a crucial role in the “capping” of telomeres through the formation of telomeric DNA:RNA G-quadruplexes [20,124,125,126]. This argument is supported by insights from a single-molecule FRET study examining the folding patterns of telomere DNA in conjunction with telomere RNA. Notably, telomere RNA exhibits a preference for the formation of DNA:RNA hybrid G-quadruplexes at the 3′ end of telomeric DNA. Furthermore, DNA G-quadruplexes is unfolded by a complementary C-rich sequence, but this effect is not observed for DNA:RNA hybrid G-quadruplexes. These findings underscore previous arguments that these hybrid G-quadruplexes protect the 3′ end of the telomeric DNA overhang, which is critical for the interaction with telomerase and other telomere-associated proteins. Unique contribution of RNA in reinforcing the structural integrity of telomeres has been evidenced. According to bioinformatic analysis, putative DNA:RNA hybrid G-quadruplex-forming sequences are abundant on both sides of the transcription start sites in the genome of warm-blooded animals [125]. Their formation is dependent on the negative supercoiling generated by RNA polymerases. Genome-wide occurrence of DNA:RNA hybrid G-quadruplexes has been also indicated [126]. The biological roles of DNA:RNA hybrid G-quadruplexes are gradually getting clearer.

## 5. Binding Proteins of Telomere DNA and RNA G-Quadruplexes 

Notably, POT1 has been demonstrated to unravel intramolecular G-quadruplexes to facilitate its binding to telomeric DNA [127,128]. The unfolding of G-quadruplexes by POT1 also impacts the capacity of telomeric sequences to undergo extension by telomerase, at least in vitro [1]. Initially, it was shown with telomerase from three distinct species of ciliated protozoa that oligonucleotides adopting antiparallel intramolecular G-quadruplex structures do not serve as favorable substrates for telomerase [129,130]. The antiparallel and hybrid G-quadruplex structures assumed by a 4-repeat oligonucleotide in K^+^ solution have been also demonstrated to be ineffective substrates for telomerase [129,130,131,132,133,134]. These G-quadruplex conformations [133,134] inhibit the binding of telomerase to the DNA, leading to a non-processive attempted extension of the DNA by telomerase [129]. POT1 can reinstate the capability of telomerase to engage with these substrates by unfolding the G-quadruplex and capturing the DNA in a linear form with a protruding tail [127]. The dynamic interplay of G-quadruplex folding and their unfolding by POT1 may thus serve as a regulatory mechanism, governing the access of telomerase to the telomere.

**Figure 6:**
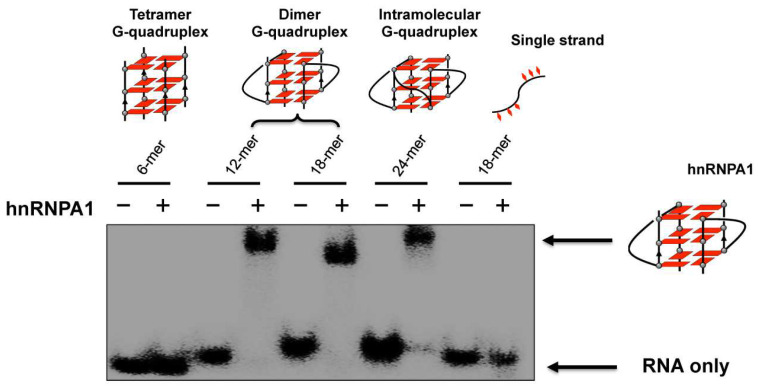
Binding of hnRNPA1 to telomere RNA G-quadruplexes of various structures. Only the RNA G-quadruplexes involving loops (the dimer and the intramolecular G-quadruplexes) bind hnRNPA1, and neither the tetramer RNA G-quadruplex without loops nor single-stranded RNA binds the protein.

**Figure 7:**
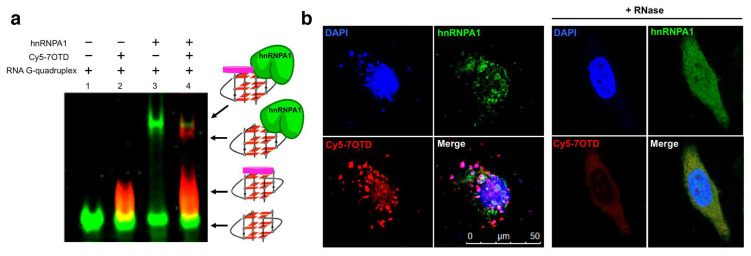
Analysis of the binding of hnRNP1 with RNA G-quadruplex using Cy5−7OTD. (**a**) Gel electrophoresis on the binding. Lane 1, RNA G-quadruplex visualized with fluorescein in green; lane 2, RNA G-quadruplex with Cy5−7OTD in red; lane 3, the mixture of RNA G-quadruplex and hnRNPA1 visualized by fluorescein in green; lane 4, the complex formation of RNA G-quadruplex with hnRNPA1 colored in red by Cy5− 7OTD. (**b**) Microscopy images of human cells incubated with Cy5−7OTD (red) and then with antibody of hnRNPA1 (green). DNA was stained by DAPI (blue fluorescence). In the merged panel, colocalization of RNA G-quadruplex (red) and hnRNPA1 (green) is apparent. As expected, the signals of RNA G-quadruplex were notably weakened when the cells were treated by RNase.

HnRNPA1 is an RNA binding protein which is involved in the telomere maintenance machinery. The binding of hnRNPA1 to telomere RNA with structure-dependence can regulate human telomere function. In Figure 6, the interactions of G-quadruplex RNA with hnRNPA1 were analyzed by gel electrophoresis in which RNA G-quadruplexes of different structures were employed [85,86]. It was demonstrated that the loops of G-quadruplex RNA are very important for interaction with hnRNPA1. Furthermore, a macrocyclic heptaoxazole molecule, 7OTD, and its fluorescent derivative, Cy5−7OTD, have been developed to bind to telomere RNA G-quadruplex and visualize its complex formation with hnRNPA1 [85]. In Figure 7a, the green band is for telomere RNA G-quadruplex. The red band of smaller mobility, observed after the addition of Cy5−7OTD, is assigned to the interaction of RNA G-quadruplex with Cy5−7OTD. Upon further addition of hnRNPA1, a new band appeared in the upper part of the gel, due to its binding with the RNA G-quadruplex. Furthermore, Cy5−7OTD was employed in Figure 7b to analyze the interactions of RNA telomere G-quadruplex with hnRNPA1 in cells. By incubating HeLa cells with this probe, the telomere RNA G-quadruplex was detected as red signals in fluorescence microscopy. On the other hand, hnRNPA1 in the cells was visualized by green fluorescence from anti-hnRNPA1 antibody-based probe. Quite importantly, the green fluorescence of hnRNPA1 and the red fluorescent foci of the RNA G-quadruplex notably overlapped each other. Consistently, the intensity of the red signal was reduced by RNase treatment, confirming that this signal came from the Cy5−7OTD bound to the RNA G-quadruplex.

The C-terminal region of TLS, a human telomere-binding protein, engages in the formation of a ternary complex with G-quadruplex DNA and RNA from human telomeres in vitro, specifically within the Arg-Gly-Gly domain [135]. Notably, TLS exhibits binding affinity to G-quadruplex telomere DNA within double-stranded regions and G-quadruplex RNA. This dual interaction plays a crucial role in regulating histone modifications associated with telomeres, influencing overall telomere length. The findings propose that the G-quadruplex serves as a scaffold for TLS, orchestrating the modulation of telomere length through histone modifications. In parallel, the N-terminal Gly/Arg-rich (GAR) domain of TRF2 demonstrates recognition capabilities for G-quadruplex RNA [136]. The G-quadruplex structure within telomere RNA emerges as a pivotal recognition element for the TRF2 GAR domain. The interaction between TRF2 GAR and telomere RNA is deemed essential for maintaining telomere stability, underlining its significance in the intricate regulatory mechanisms governing telomeric function.

Alternative lengthening of telomeres (ALT), a telomerase-independent mechanism for maintaining telomeres, is orchestrated primarily through break-induced replication (BIR) [137,138]. The human protein RAD52 plays a key role in promoting ALT by facilitating the formation of D-loops. However, it is noteworthy that ALT can also occur via a RAD52-independent BIR pathway. In the absence of RAD52, telomere RNA contributes significantly to ALT activity by forming dynamic telomeric R-loops. These R-loops, in turn, augment the presence of G-quadruplex structures at telomeres. The increased G-quadruplex formation enhances ALT, even in cases where telomeric RNA is depleted. This observation suggests that G-quadruplexes operate downstream of R-loops, playing a pivotal role in promoting break-induced replication and contributing to the ALT process.

Telomeric RNA exhibits the ability to adopt a G-quadruplex structure and engage in interactions with the chromatin remodeler ATRX [139]. In this interaction, telomere RNA acts as a regulator, preventing ATRX from binding to subtelomeric regions. Instead, it facilitates the promotion of G-quadruplex formation in proximity to transcription start sites (TSS) that are distant from telomeres. Upon depletion of telomere RNA, there is an observed increase in ATRX binding at TSS, leading to the unwinding of G-quadruplex DNA and subsequent repression of gene expression. This unveils an epigenetic regulatory mechanism by which telomere RNA serves to sequester ATRX, thereby preserving DNA G-quadruplex structures and influencing gene expression patterns.

## 6. Applications of G-Quadruplexes

**Figure 8:**
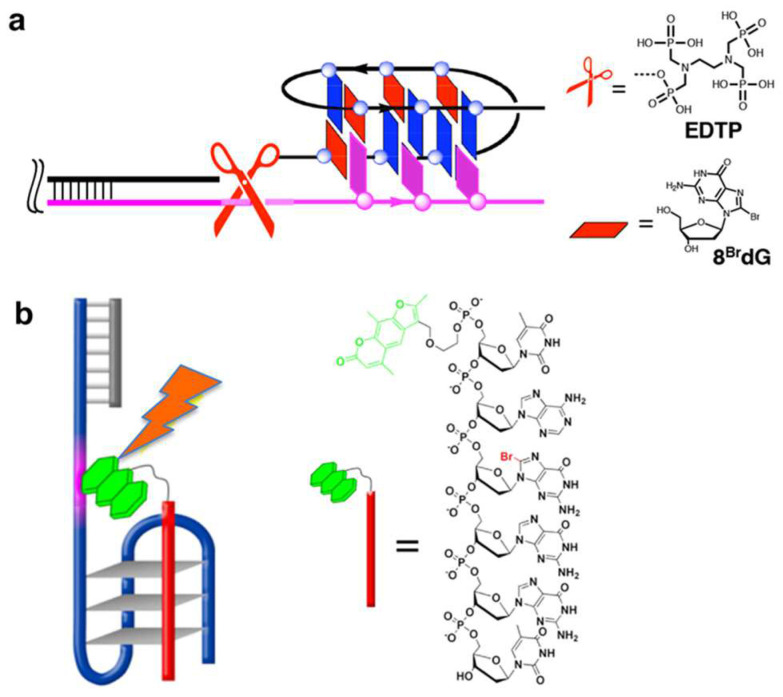
(**a**) Telomere DNA specific cleavage by G-quadruplex formation. The structures of ligand EDTP and 8^Br^dG are shown. (**b**) Photochemical inhibition of telomerase by targeting human telomere DNA with a 6-mer DNA (red bar) through G-quadruplex formation. The 6-mer DNA bears a psoralen (green) at the 5′-end.

Unique structural restraints of G-quadruplexes are effectively employed as scaffolds for specific biological and/or chemical reactions. Freedoms of structural design of the systems are highly advantageous for these applications. In terms of the formation of DNA G-quadruplex, a chemical method to selectively cleave human telomere DNA was developed [83]. In Figure 8a, 16-mer DNA of three-repeat human telomere sequence (^Br^GGGTTA^Br^G^Br^GGTTAGGGT) binds to a telomere DNA substrate through the formation of dimeric G-quadruplex. To the 5′-end of this 16-mer DNA, an ethylenediamine-*N,N,N’,N’*-tetrakis(methylenephosphonic acid) (EDTP) as metal-binding group is conjugated. Upon the addition of Ce^IV^(NH_4_)_2_(NO_3_)_6_, Ce^IV^ ion (a catalyst for DNA hydrolysis) is bound to the EDTP, and placed near the phosphodiester linkage(s) in the telomere DNA substrate. As a result, only telomere DNA is picked up from reaction mixtures through G-quadruplex formation, and cleaved through hydrolytic pathway. 

A similar strategy was employed to design a photo-controllable inhibitor of telomerase, which is vigorously expressed in cancer cells (Figure 8b) [140]. To the 5′-end of 6-mer DNA (TA^Br^GGGT), a photosensitive dye psoralen (green colored) was conjugated. This photo-crosslinking reagent is a linear furanocoumarin, and selectively reacts with pyrimidine bases (T and C) for crosslinking. This psoralen-conjugated 6-mer DNA binds to telomere DNA through the formation of intermolecular G-quadruplex. Upon photo-irradiation, the psoralen crosslinked the telomere DNA with the 6-mer DNA. As the result, the duplication of telomere DNA by telomerase was sterically inhibited, triggering the death of cancer cells. These two results apparently show that G-quadruplex formation serves as an efficient approach to target human telomere for its effective transformation.

Alternatively, the formation of an intermolecular G-quadruplex presents a novel mechanism to regulate the efficiency of translation [141]. A G-rich sequence (5′-GGGCCCGGG-3′) was introduced to the 5′-UTR of mRNA, and a telomere related G-rich RNA (5′-GGGUUAGGG-3′) was employed as on-off controller of translation. An enhanced green fluorescent protein reporter (EGFP1) was employed. Without the addition of the short telomere RNA, the 5′-UTR of mRNA was in the normal state, and EGFP1 protein was efficiently expressed to emit green fluorescence. Upon the addition of the telomere short RNA. However, EGFP1 was hardly expressed. Apparently, the mRNA translation was inhibited by the formation of RNA G-quadruplex between the short telomere RNA and the G-rich sequence in the 5′-UTR. The short telomere RNA successfully worked as an on-off controller of translation.

## 7. Future Prospects and Conclusions

Telomere biology has come a long way in the past 70 years, from the observation that chromosomes require protection to the award of the 2009 Nobel Prize for the discovery of telomerase and the effects of telomere shortening on cells. Functional telomeres have been experimentally implicated in a number of molecular cell processes. Molecular components of telomeres, including terminal double- and single-stranded telomeric DNA, protein complex shelterin, and telomerase form physical states which are associated with functional capping and uncapping. In this decade in particular, structural information of DNA G-quadruplexes and RNA G-quadruplexes has been rapidly accumulated, deepening our understanding on the biological roles of telomeres. Pre-clinical experiments suggested that telomere is an encouraging target for cancer therapy. The findings of telomere RNA molecules open new doors to a better understanding of the complicated functions of telomeres. These telomere RNAs are bound by a complicated set of proteins [142]. Furthermore, telomeric RNA G-quadruplex and DNA G-quadruplex show homogeneous dimerization and mutual association. Telomere RNA, a newly emerging player, may be useful for cancer treatment, since these RNA molecules accompanies malignant transformation. Thus, telomere RNA G-quadruplex structures should be also a valuable target for anticancer agents [1]. Stabilization of telomere RNA G-quadruplex structures by small molecules can lead to telomerase inhibition. All of these results confirm that there are crucial needs to revisit the structural and functional mechanisms of telomeres.

**Figure 9:**
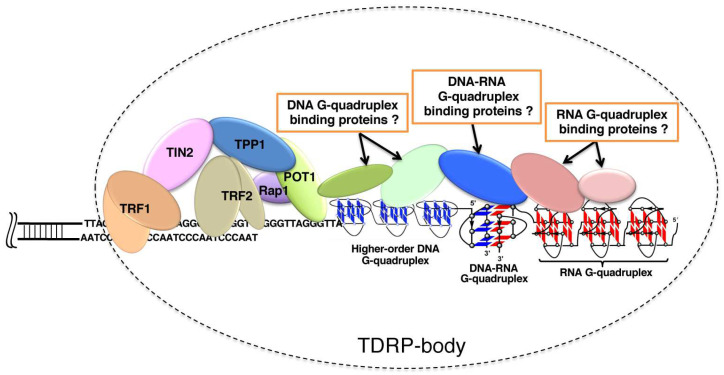
A schematic view of human telomere end: telomere DNA, telomere RNA, and related-proteins in the TDRP body. Proteins can recognize DNA telomere G-quadruplex, RNA telomere G-quadruplex and hybrid DNA:RNA G-quadruplex. The TDRP body at the ends of chromosomes provides a protective structure. Note that some of the contents could be speculative, and have not necessarily been firmly established.

The current status of our understanding on telomere science is schematically depicted in Figure 9. Telomere DNA, telomere RNA, and related-proteins construct TDRP body (telomeric DNA, RNA and proteins body), such as P bodies for mRNA surveillance, mRNA decay, RNA-mediated silencing and translational control [143]. Telomere capping is promoted by the binding of POT1 protein to single-strand telomere DNA [84]. Many evidences show that telomere RNAs are associated with various elements in the telomere. Through the formation of telomeric DNA:RNA G-quadruplexes, telomere RNA interacts with telomeric DNA for the ‘‘capping’’ of telomere. Moreover, telomeric RNA is a structural component of the telomere nucleoprotein complex, and is also bound to the telomere through interactions with Shelterin components such as TRF1 and TRF2. Three domains of TRF2 interact with telomeric RNA including the amino-terminal basic domain (GAR) [144]. Telomeric RNA further interacts with the ORC1 region that associates with TRF2 GAR. Interestingly, the TRF2 GAR has a relatively high affinity towards G-rich RNA capable of forming G-quadruplex structures, indicating that the recognition mainly depends on G-quadruplex structure, rather than a specific sequence [85]. Telomeric RNA promotes POT1 binding to telomeric DNA by removing hnRNPA1 [84]. Thus, the accumulation of telomeric RNA is helpful for the formation of TDRP body and promotes telomere capping. It was also shown that acridine ligands notably change the UUA loop conformation of RNA G-quadruplex [145]. The resultant stabilization of G-quadruplexes should facilitate the formation of TDRP bodies, in which higher-order telomere structures are satisfactorily capped to accomplish homeostatic functions. 

Furthermore, it should be noted that newly discovered telomere RNA may provide a solution to “the end replication problem” in which a protruding single-stranded 3′-DNA end causes a progressive shortening of telomeric DNA at each round of DNA replication [146,147]. In normal cells, telomere RNA can act as a template for telomere elongation synthesis by reverse transcriptase. Support for this hypothesis comes from the finding that telomere RNAs are significantly downregulated in various types of human cancers, compared to normal tissues. In normal cells, the reverse transcription pathway is involved in the maintenance of telomere length, whereas telomerase is mainly responsible for telomere length maintenance in cancer cells.
